# Evaluation of the Effects of Different Dietary Patterns on Breast Cancer: Monitoring Circulating Tumor Cells

**DOI:** 10.3390/foods10092223

**Published:** 2021-09-19

**Authors:** Xiuxiu Wang, Xiaoyu Liu, Zhenzhen Jia, Yilun Zhang, Shuo Wang, Hongyan Zhang

**Affiliations:** 1 Shandong Provincial Key Laboratory of Animal Resistance Biology, Institute of Biomedical Sciences, Key Laboratory of Food Nutrition and Safety of Shandong Normal University, College of Life Science, Shandong Normal University, Jinan 250014, China; Xiux_wang@163.com (X.W.); liuxiaoyu20200145@163.com (X.L.); jiazhenzhen@sdnu.edu.cn (Z.J.); zhangyilun0@163.com (Y.Z.); 2 School of Medicine, Nankai University, Tianjin 300457, China

**Keywords:** dietary patterns, breast cancer, tumor metastasis, circulating tumor cells, *in vivo* capture

## Abstract

The occurrence and development of breast cancer are closely related to dietary factors, especially dietary patterns. This study was to investigate the effects of dietary patterns on the process of tumor metastasis by *in vivo* circulating tumor cell (CTC) capture strategy and monitoring changes of CTC numbers in breast tumor mice model. Meanwhile, the effects of different dietary patterns on the development of lung metastases of breast cancer and the volume and weight of carcinoma in situ were investigated. In this study, the increase in the number of CTCs was significantly promoted by dietary patterns such as high-salt diet, high-sugar diet, and high-fat diet, while it was delayed by ketogenic diet, low-fat diet, low-protein diet, diet restriction, and Mediterranean diet. These results indicated that the *in vivo* capture and detection of CTCs provides a convenient method for real-time cancer metastasis monitoring, and through in-depth study of the effects of different dietary patterns on tumor growth and metastasis, it can expand a new horizon in future cancer treatments.

## 1. Introduction

Breast cancer is one of the most common malignancies that plague women worldwide [1]. The majority of patients deaths are mainly due to cancer metastasis, especially lung metastases [2]. Dietary habits and lifestyle-related factors are key determinants of cancer risk and metastasis, and most (80–90%) cancers are caused by external environmental factors, of which 30–40% are directly related to diet. Seventy percent of the causes of sporadic breast cancer come from environmental factors, especially dietary factors [3,4].

Research showed that an unhealthy dietary pattern exacerbates cancer metastasis, and a healthy dietary pattern increases the suppressive effect on cancer metastasis [5,6], so it is important to choose a healthy dietary pattern. Additionally, when a healthy diet is combined with chemotherapy, immunotherapy, or other treatments, it may be a promising strategy to improve the effectiveness of treatment, preventing the acquisition of resistance and reducing side effects, and it is expected to have significant medical, social, and economic impacts [7]; hence, this paper explores the effects of different dietary patterns on the metastasis progression of breast cancer. 

**Scheme 1:**
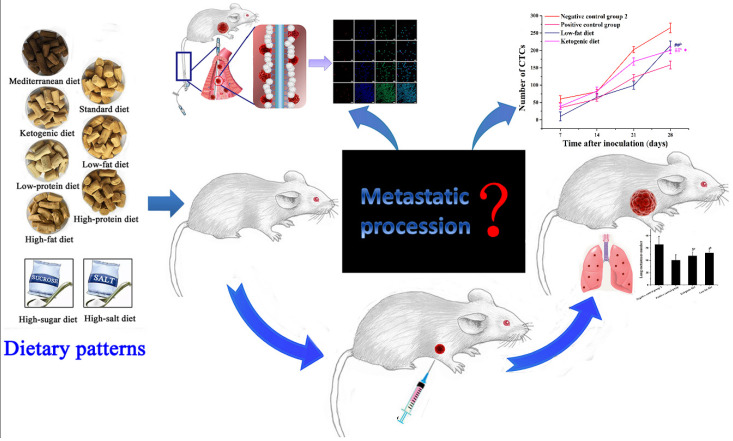
A Schematic model of effects of different dietary patterns on breast cancer metastasis based on *in vivo* capture of CTCs. ^#b^ *p* < 0.05 and ^##b^ *p* < 0.01 compared with negative control group 2. * *p* < 0.05 compared with that of positive control group.

Currently, the research on breast cancer with different dietary patterns is mainly focused on *in vivo* tumor experiments [8]. However, the traditional experiments can only reveal the final results of metastasis and cannot monitor the process of tumor metastasis *in vivo* in real-time, which is like a black box (Scheme 1). To solve this problem, our group developed a method for capturing circulating tumor cell (CTC) *in vivo* [9]. CTCs are tumor cells that are break off from primary tumors, flowing in the blood-stream to other organs where they initiate metastatic growth [10]. CTCs also provide a sensitive indicator for evaluating different dietary patterns on breast cancer progress.

In this study, 10 different dietary patterns including standard diet, high-salt diet, high-sugar diet, high-fat diet, low-fat diet, high-protein diet, low-protein diet, ketogenic diet, diet restriction, and Mediterranean diet were selected as the research subjects. While studying the effects of different dietary patterns on the growth of carcinoma in situ and lung metastases, we focused on the *in vivo* CTCs capture to assess the cancer metastasis in real-time to evaluate the effects of different dietary patterns on tumor metastasis. This study provided valuable suggestions for people to eat properly to achieve the purpose of cancer prevention or treatment, and this is an important report evaluating the effects of multiple dietary patterns on breast cancer metastasis simultaneously.

## 2. Materials and Methods

### 2.1. Materials

EpCAM rabbit polyclonal antibody was obtained from Bioss Biological Technology Co., Ltd. (Beijing, China). Anti-CD45-FITC specific for leukocytes and 4′,6-diamidino-2-phenylindole (DAPI) were purchased from Invitrogen (Carlsbad, CA, USA). ROX-muc1 aptamer probes (5′-GCAGTTGATCCTTTGGA TACCCTGG-3′) were synthesized by Sangon Biotech Co., Ltd. (Shanghai, China) [11]. Paraformaldehyde and erythrocyte lysate were obtained from Solarbio Science & Technology Co., Ltd. (Beijing, China). Polydimethylsiloxane (PDMS) prepolymer and curing agent were obtained from Dow Corning (Midland, TX, USA). Roswell Park Memorial Institute (RPMI) 1640 medium was purchased from Zhejiang Tianhang Biotechnology Co., Ltd. (Zhejiang, China).

### 2.2. Cell Culture

The mouse breast cancer cell line 4T1 was obtained from the Cell Bank of the Chinese Academy of Sciences (Shanghai, China), which were cultured in RPMI 1640 medium supplemented with 10% fetal bovine serum (FBS) and 1% penicillin/streptomycin double antibiotic solution, and the cells were placed in a 37 °C, 5% CO_2_ carbon dioxide cell incubator. The digested 4T1 cells were centrifuged to make a cell suspension, and a cell suspension with a concentration of 10^6^ cells/mice was inoculated into the underarms of mice. 

### 2.3. Construction of 4T1 Breast Cancer Tumor Mouse Model

Female BALB/c mice that were four weeks old and weighed 18–24 g were purchased from Beijing Vital River Laboratory Animal Technology Co., Ltd. (Beijing, China). Mice were randomly grouped according to their weight (six in each group). The mice were housed in a standard animal house and a clean environment. The temperature of the mouse room was controlled at 18–20 °C. The light and dark cycle was maintained for 12:12 h, kept ventilated, and fed for nine weeks. The animal studies were performed in accordance with ethical regulations and approved by the institutional animal care and use committee of Shandong Normal University, Jinan, China.

To evaluate and compare the effects of different dietary patterns objectively, dietary habits from childhood to youth were simulated. Subsequently, dietary interventions were started from the age of 4 weeks. As the mice in the high-sugar diet and high-salt diet groups may have difficulty controlling the amount of food due to preference problem, each mouse was administered intragastrically with 200 μL of a different dose solution each day. high-sugar diet was intragastrically administered with 5500 mg (kg·d)^−1^ sucrose [12], and high-salt diet was intragastrically administered with 5250 mg (kg·d)^−1^ NaCl [13,14]. The negative control group 1 was intragastrically administered with 200 μL of physiological saline daily and the positive control group was intragastrically administered paclitaxel with dose of 40 mg (kg·d)^−^^1^ [15,16]. Compared with that of the negative control group 2, mice of the diet restriction group needed to reduce their food supply by 30% daily, and mice of the other groups were given ad libitum diet. The component content corresponding to the different diet patterns of the mouse 4T1 allograft model was shown in Table 1. When the mice were 8 weeks old, 4T1 cells were inoculated subcutaneously in the axillary of the right forelimbs of the mice. The volume of carcinoma in situ was calculated according to the following formula: volume = 0.5 × (width)^2^ × (length) [17]. Besides, the volume of carcinoma in situ was measured every four days. On the 35th day, all mice were sacrificed, the lungs and tumors were dissected out, and the number of lung metastases of breast cancer, tumor volume, and weight were recorded.

### 2.4. Preparation of Functionalized Indwelling Needle

The method of coating the EpCAM antibody on the indwelling needle were referred to previous research after some modifications [27]. The prepolymer was mixed with the curing agent at a ratio of 10:1 to make a PDMS gel, and then degassed in a vacuum for 40 min. After removing the air bubbles, all the indwelling needle hoses were immersed in PDMS gel and dried at 85 °C for 90 min. The indwelling needle tubes were incubated in EpCAM antibody solution (100 µg·mL^−1^) overnight at 4 °C.

### 2.5. Capture of CTC In Vivo

We evaluated the effects of different dietary patterns on tumor metastasis by capturing CTCs every 7 days after implanting mice with 4T1 cells (4 times in total capture). The functionalized indwelling needle was inserted into the tail vein of the mouse, and the hose portion of the indwelling needle was left in the caudal vein. After 2 h, the cells adhered to the indwelling needle tube were washed into the microtiter plate with 200 µL of PBS and left for 30 min.

### 2.6. Immunofluorescence Staining and Counting of CTCs

Fixative (50 µL) was added to aspirate PBS, and fixed for 15 min (25 °C). Permeabilization solution (50 µL) was added, and the cells were permeated for 25 min (25 °C). After the membrane permeation was aspirated, 200 µL of erythrocyte lysate was added and lysed for 5 min (4 °C). Anti-CD45-FITC solution (30 µL, 5 µg·mL^−1^) was added to each well according to the group and incubated at 37 °C in the dark for 1.5 h to stain the leukocytes. ROX-muc1 aptamer (75 µL, 0.05 µM) was added and incubated at 4 °C in the dark for 1.5 h to stain the CTCs. Nuclear staining was then performed by incubating with 50 µL of DAPI (0.1 µg·mL^−1^) for 10 min in the dark. Finally, the LEICA automatic inverted fluorescence microscope was used to observe and identify CTCs.

### 2.7. Hematoxylin and Eosin (H&E) Staining

Lung tissues were fixed with 4% paraformaldehyde. The fixed tissues were embedded, sectioned, and stained with H&E, and the images were scanned with digital slice scanner.

### 2.8. Statistical Analysis

These values are provided as mean ± standard deviation. One-way analysis of variance (ANOVA) was used to test the significance of the difference between the averages of each group, and pairs of variables were compared using the Statistical Product and Service Solutions (SPSS). The threshold of statistical difference assumes that *p* > 0.05 indicates that the difference is not significant; *p* < 0.05 indicates that the difference is significant; *p* < 0.01 indicates that the difference is extremely significant.

## 3. Results

### 3.1. Effects of Different Dietary Patterns on the Growth of Carcinoma In Situ

**Figure 1:**
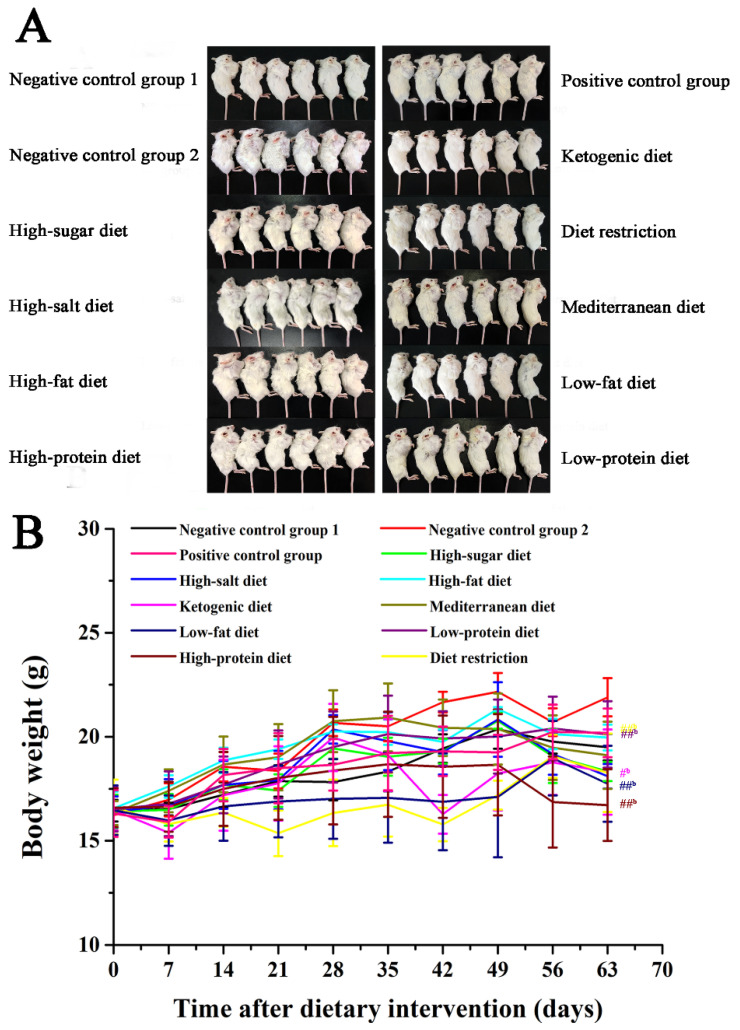
Effects of different dietary patterns on body weight. (**A**) Images of tumors in mice, *n* = 6. (**B**) Mice weights were recorded every 7 days. ^#b^ *p* < 0.05 and ^##b^ *p* < 0.01 compared with negative control group 2.

**Figure 2:**
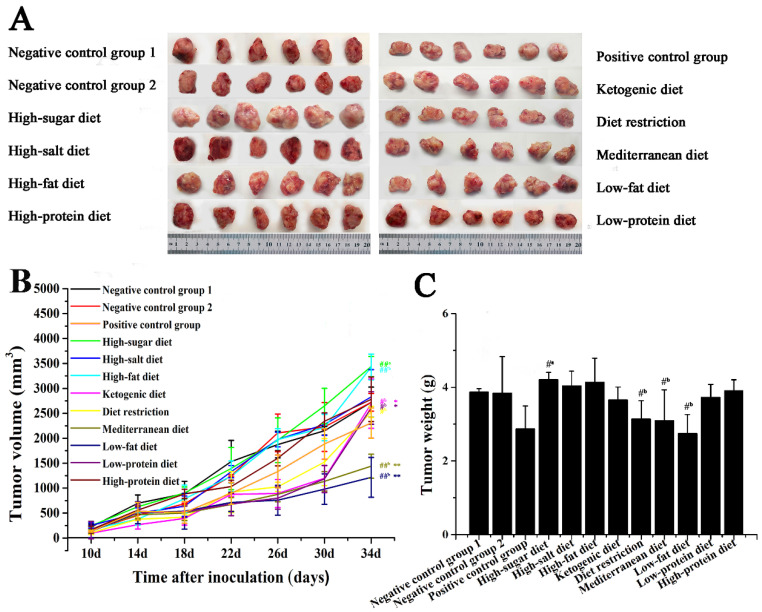
Effects of different dietary patterns on tumor in situ. (**A**) Image of a mouse tumor removed after dissection. (**B**) Tumor volume of mice was recorded every 4 days. (**C**) Tumor weight of mice removed after dissection. ^#a^ *p* < 0.05 and ^##a^ *p* < 0.01 compared with negative control group 1. ^#b^ *p* < 0.05 and ^##b^ *p* < 0.01 compared with that of negative control group 2. * *p* < 0.05 and ** *p* < 0.01 compared with that of positive control group.

To evaluate the effects of different dietary patterns on the growth of carcinoma in situ in 4T1-xenograft mice, mice were given specific dietary interventions starting at 4 weeks of age for 9 weeks. Due to different dietary patterns, the weight of mice showed a certain difference. As shown in Figure 1A,B, the weight of mice in the high-protein diet, low-fat diet, and diet restriction groups was significantly lighter than that of the negative control group 2 (*p* < 0.01). In Figure 2A,B, the tumor volume of high-sugar diet group was significantly larger than that of the negative control group 1, the tumor volume of the high-fat diet groups was significantly larger than that of the negative control group 2 (*p* < 0.01). Compared with that of the negative control group 2, the low-fat diet, Mediterranean diet, diet restriction, low-protein diet, and ketogenic diet groups all inhibited tumor growth to vary degrees (*p* < 0.05), while the inhibition of low-fat diet and Mediterranean diet groups was much stronger (*p* < 0.01). The tumor volume of the low-fat diet and the Mediterranean diet groups was significantly smaller than that of the positive control group (*p* < 0.01).

Tumors were removed at 35 days after the inoculation. The tumor weight of the low-fat diet, diet restriction, and Mediterranean diet groups was less than negative control group 2 (*p* < 0.01), the difference was not significant compared with that of the positive control group (*p* > 0.05). The tumor weight of the high-sugar diet group was greater than that of negative control group 1 (*p* < 0.05), which promoted the growth of carcinoma in situ. High-sugar diet group increased tumor weight to 4.20 ± 0.16 g (*p* < 0.05) from weight of 3.87 ± 0.09 g in the negative control group 1 (Figure 2C), and the tumor volume increased from 2723.51 ± 302.60 mm^3^ to 3347.58 ± 202.95 mm^3^ (*p* < 0.01) (Figure 2B), which all showed that the high-sugar diet promotes the growth of carcinoma in situ.

### 3.2. Effects of Different Dietary Patterns on Metastasizing Tumors of Lung

**Figure 3:**
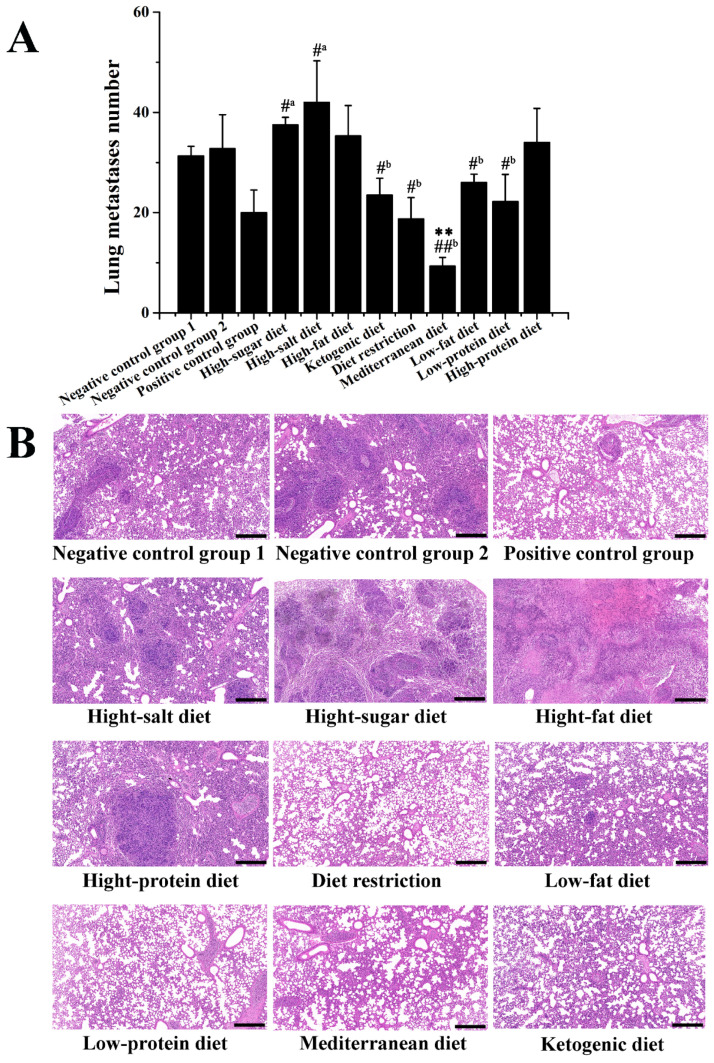
Effects of different dietary patterns on lung metastasis of 4T1-xenograft mice. (**A**) Number of lung metastasis. ^#a^ *p* < 0.05 compared with that of negative control group 1. ^#b^ *p* < 0.05 and ^##b^ *p* < 0.01 compared with that of negative control group 2. ** *p* < 0.01 compared with that of positive control group. (**B**) HE-staining of lung tissues (scale was 400 μm).

As shown in Figure 3A, compared with that of negative control group 2, the five dietary patterns of the Mediterranean diet, diet restriction, low-protein diet, ketogenic diet, and low-fat diet all have different degrees of delay in lung metastasis. The number of lung metastasis in the Mediterranean diet and diet restriction groups was significantly lower than that in that of negative control group 2 (*p* < 0.01), and the number of lung metastasis in the Mediterranean diet group was significantly lower than in that of the positive control group (*p* < 0.01). Contrarily, the number of metastasizing tumors of lung in the high-sugar diet and high-salt diet groups was higher than that in the negative control group 1 (*p* < 0.05). According to HE-staining results in Figure 3B, the pulmonary alveoli were destructed, and inflammatory cell infiltration was observed in the negative control group 1 and negative control group 2 mice lung tissues. Compared to that of the negative control group 2, the Mediterranean diet, diet restriction, low-protein diet, ketogenic diet, and low-fat diet treatments improved the pulmonary alveoli tissue morphology and suppressed lung metastasis. Compared to that of the negative control group 1, the high-sugar diet and high-salt diet treatments exacerbated lung tissue damage and metastasis.

### 3.3. Effects of Different Dietary Patterns on CTCs

**Figure 4:**
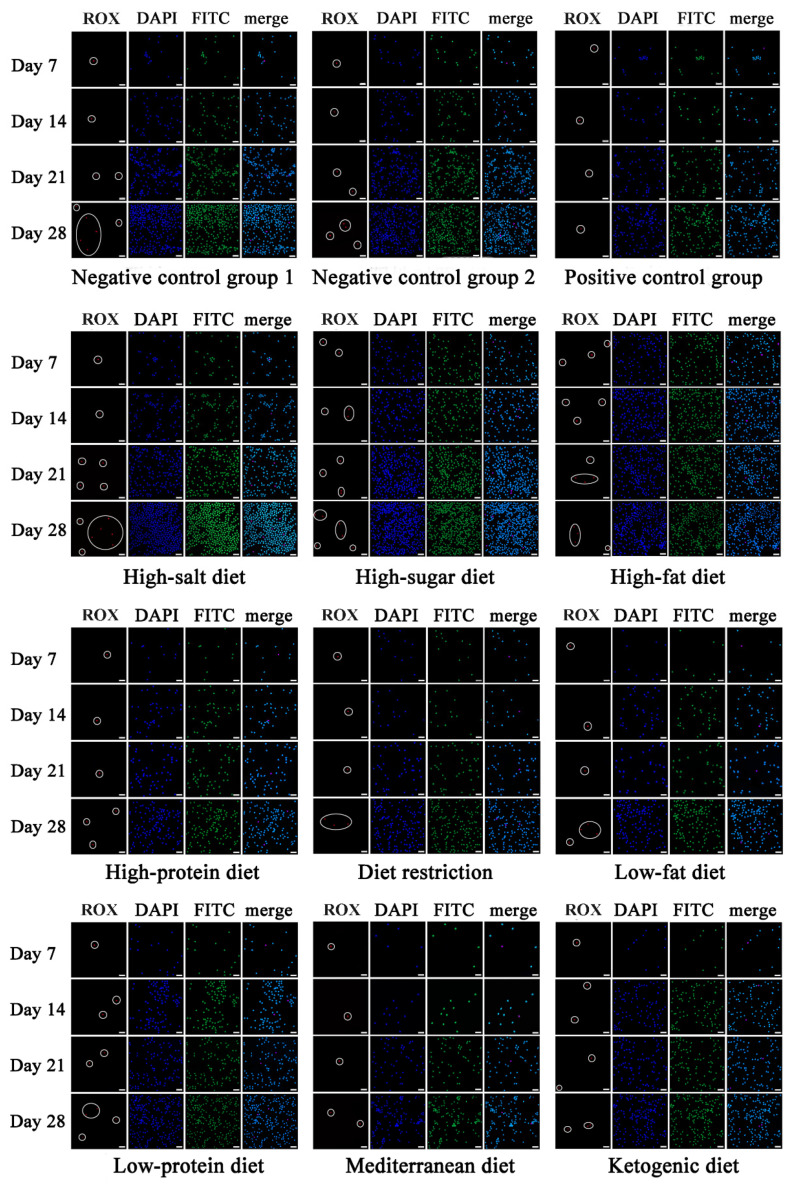
Fluorescent images of CTCs captured *in vivo* for different dietary patterns. CTCs were marked by circle (scale was 25 μm). ROX, acceptor fluorophore (red).

**Figure 5:**
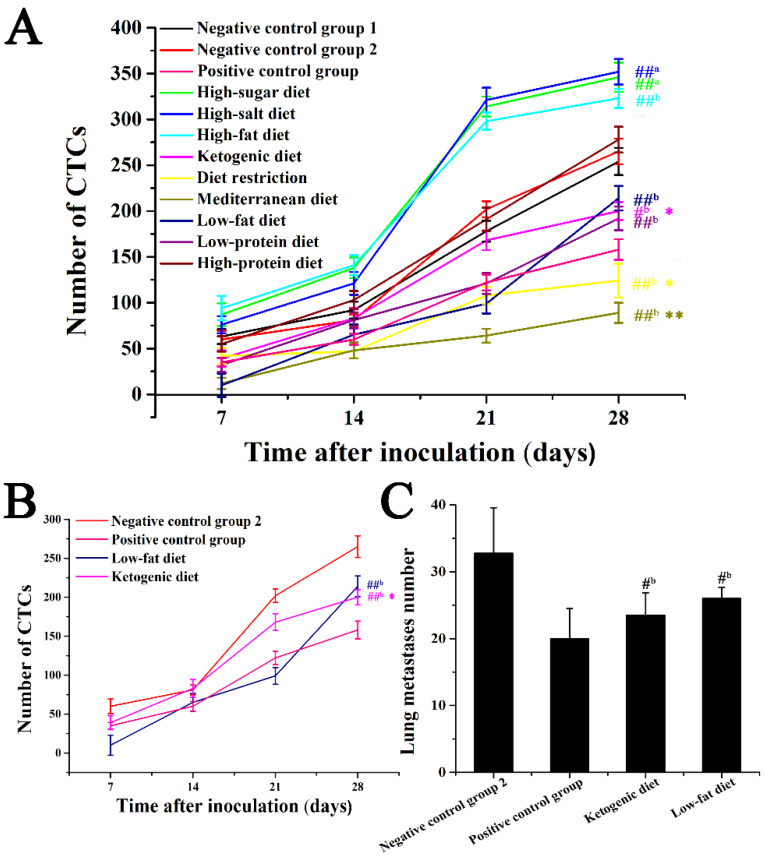
Effect of different dietary patterns on CTC numbers. (**A**) CTC numbers of different dietary patterns during cancer progression. (**B**) CTC numbers of Mediterranean diet group and high-salt diet group during cancer progression. (**C**) Numbers of lung metastatic cancers of Mediterranean diet group and high-salt diet group during cancer progression. ^##a^ *p* < 0.01 compared with that of negative control group 1. ^#b^ *p* < 0.05 and ^##b^ *p* < 0.01 compared with that of negative control group 2. * *p* < 0.05 and ** *p* < 0.01 compared with that of positive control group.

Previous research by our group explored the effective capture of CTCs on mouse tumor models with functional indwelling needles [9]. This method can capture CTC in the blood on the seventh day after tumor inoculation, which can detect the occurrence of tumor metastasis in early cancer. In this study, the effects of different dietary patterns on breast cancer metastasis were evaluated by detecting CTC numbers at different time points during breast cancer metastasis and according to the trend of the number of CTCs (Figure 4). According to the results of *in vivo* capture of CTC in a mouse 4T1-xenograft model, CTCs can be captured on the seventh day after vaccination. As shown in Figure 5A, the change in the number of CTCs was closely related to cancer metastasis, and CTC numbers showed an upward trend with the extension of the duration of cancer. The number of CTCs in the five dietary patterns including low-fat diet, ketogenic diet, low-protein diet, diet restriction, and Mediterranean diet was lower than that of negative control group 2 (*p* < 0.05), among which the Mediterranean diet and diet restriction groups exhibited stronger anti-metastasis effect (*p* < 0.01), and showed more effective anti-metastasis effect than that of the positive control group (*p* < 0.05). Low-protein diet group had comparable antimetastatic effects to that of the positive control group (*p* > 0.05). In Figure 5B,C, compared with that of negative control group 2, the increase in a number of CTCs in the low-fat diet and ketogenic diet groups was also delayed (*p* < 0.05), however, the antimetastatic effect was worse than that of the positive control group. In the process of tumor metastasis, different dietary patterns have different effects on the number of CTCs. For example, the Mediterranean diet group showed a more effective anti-metastasis effect over the course of cancer metastasis than that of the positive control group, but the low-fat diet group showed a more rapid increase in the number of CTCs in the later stages of cancer, and the antimetastatic effect was worse compared with that of early and middle stages. Based on the detection of the number of CTCs, the effects of these different dietary patterns on cancer metastasis can be monitored in real-time during the metastasis process.

## 4. Discussion

The effects of dietary patterns on cancer obtained significant attention. The current cancer research about diets mainly focuses on *in vitro* experiments and *in vivo* tumor experiments. Despite the development of cancer treatments, tumor metastasis remains a major driver of death in cancer patients [28] because it was difficult to monitor cancer metastasis early in the cancer using conventional monitoring methods. Furthermore, in routine experiments, only assessing the number of lung metastatic cancers is not enough to assess the entire process of tumor metastasis, and real-time monitoring cannot be performed [29]. Compared with that of the in vitro CTC-enrichment method, the method of capturing CTC *in vivo* improves the capture efficiency and sensitivity [9]. Therefore, the capture and detection of CTCs were used to monitor the change in the number of CTCs in real-time to evaluate effects of different dietary patterns on breast cancer metastasis.

Breast cancer is the most common malignancy that plagues women worldwide. Clinically, breast cancer is usually treated with surgery, radiotherapy or chemotherapy [30]. Those therapies do not fundamentally reduce the risk of breast cancer, and chemotherapy drugs often have multiple toxic and side effects and produce different degrees of drug resistance. Many cancer patients experience acute or long-term side effects during cancer treatment, and it may profoundly affect their quality of life [31]. Diet is an important factor affecting the occurrence and development of breast cancer, and different dietary patterns are closely affecting the progress of cancer [32,33]. Due to the strong survival ability, migration and invasion characteristics of tumor cells are the main causes of tumor morbidity and mortality, so the study of different dietary patterns on the process of tumor metastasis has very important significance. According to the results of *in vivo* CTC capture of mouse tumor models, some interesting results were found. In this study, adherence to the Mediterranean diet was shown to be inversely associated with development of situ tumor and lung metastasis in 4T1-xenograft mice. The increased quantity and quality of fish meal, mixed fruit, vegetable powder, olive oil, and red wine contained by the Mediterranean diet could contribute to antitumor effects. In addition, in Figure 5C, there was no significant difference in the amount of lung metastatic cancers between ketogenic diet group and low-fat diet group. However, the growth trend of the number of CTCs at different points in time was diverse (Figure 5B). Low-fat diet group showed a significant inhibitory effect on the number of CTCs in the first 21 days after vaccination, but after 21 days, the inhibition of metastasis was less than that in the early and middle stages. In addition, for ketogenic diet group, the increase in CTCs after 21 days was significantly delayed, but the early inhibition of metastasis was not significant. Different dietary patterns play a role in cancer progression at different points in time, which may be the key substances that play a role at different points in time, thus regulating different signaling pathways. Therefore, it is also possible to delve into the role of different ingredients in various dietary patterns, or to combine different dietary patterns with conventional drugs to improve treatment.

In this study, several common dietary patterns were extensively screened, and the effects of multiple dietary patterns on the cancer process were compared at the same time. *In vivo* experiments to simulate the effects of different dietary patterns on the tumor metastasis process were more specific and intuitive. The results could provide many valuable suggestions for people’s healthy diet.

## 5. Conclusions

In summary, this study is based on *in vivo* capture of CTCs to evaluate the effects of multiple dietary patterns on the process of breast cancer through monitoring the number of CTCs in real-time. Ten different dietary patterns designed in our experiment significantly promote or inhibit lung metastasis of breast cancer at different stages. Therefore, in-depth exploration of the role of different ingredients in various diet patterns may provide new insights into breast cancer therapy. Meanwhile, the trend of the CTC numbers can reflect the dynamic changes of process of breast cancer, which can be used for the prognosis evaluation of different dietary patterns. This study provides a new vision and lays the foundation for future breast cancer treatment and prognosis monitoring by changing diet patterns.

## Figures and Tables

**Table 1:** Different dietary patterns and component content of mice tumor models.

	Dietary Patterns and Component Content (g/100 g)							
Composition	Standard Diet	KD	HPD	LPD	HFD	LFD	DR	MD
Fish meal	-	-	-	-	-	-	-	6.00
Soybean meal	26.00	25.00	38.00	9.50	25.00	26.00	26.00	20.00
Corn starch	25.00	-	20.00	35.00	13.65	27.00	25.00	8.90
Wheat flour	26.00	-	20.00	32.50	18.56	29.20	26.00	9.00
Wheat bran	4.00	4.00	3.00	4.00	2.18	4.00	4.00	6.00
Mixed Fruit and Vegetable Powder	-	-	-	-	-	-	-	30.00
Maltodextrin	0.50	-	0.50	0.50	2.50	0.50	0.50	0.50
Sucrose	10.00	-	10.00	10.00	14.00	10.00	10.00	10.00
Soybean oil	7.00	70.0	7.00	7.00	1.09	1.80	7.00	-
Lard	-	-	-	-	21.50	-	-	-
Olive oil	-	-	-	-	-	-	-	7.00
Salt	0.40	0.30	0.40	0.40	0.40	0.40	0.40	0.40
L-cystine	0.50	0.27	0.50	0.50	0.55	0.50	0.50	0.50
Mineral mixture	0.20	0.14	0.20	0.20	0.26	0.20	0.20	0.20
Vitamin mixture	0.20	0.19	0.20	0.20	0.20	0.20	0.20	0.30
Choline Bitartrate	0.20	0.10	0.20	0.20	0.11	0.20	0.20	0.20
Red wine	-	-	-	-	-	-	-	1.00
References	[18]	[18]	[19]	[20,21,22]	[18]	[23,24]	[18]	[25,26]

Standard diet: negative control group 1; high-salt diet; high-sugar diet; negative control group 2; positive control group. KD, ketogenic diet; HPD, high-protein diet; LPD, low-protein diet; HFD, high-fat diet; LFD, low-fat diet; DR, diet restriction; MD, Mediterranean diet.

## Data Availability

Not applicable.
